# Minimum Dietary Diversity for Women: precision of national surveys and accuracy of brief data collection instruments

**DOI:** 10.1186/s40795-025-01065-7

**Published:** 2025-05-27

**Authors:** Giles T. Hanley-Cook, Simone M. Gie, Juan Pablo Parraguez, Bridget A. Holmes

**Affiliations:** Food and Nutrition Division (ESN), Food and Agriculture Organization of the United Nations (FAO), Viale delle Terme di Caracalla, Rome, 00153 Italy

**Keywords:** Accuracy, Demographic and Health Surveys, Dietary diversity, Diet Quality Questionnaire, Gallup World Poll, Measurement agreement, Minimum Dietary Diversity for Women, Precision

## Abstract

**Background:**

Minimum Dietary Diversity for Women (MDD-W) has been identified as a promising indicator for monitoring diets globally. MDD-W questionnaires have been integrated into, amongst others, the Demographic and Health Surveys (DHS) and the Gallup World Poll (GWP). Disagreement between estimates of healthy diet metrics for the same country collected in the same year is not desirable.

**Objectives:**

To determine the measurement agreement of MDD-W estimates collected through DHS and GWP (i.e., precision), to assess the impact of survey characteristics on potential discordance, and to examine the comparative validity of the brief data collection instruments used to estimate MDD-W (i.e., accuracy).

**Methods:**

Using meta-data from DHS and GWP, we quantified the percentage points (pp) difference in food group consumption and MDD-W prevalence. Furthermore, we qualitatively examined the differences of four survey characteristics: food groups and sentinel foods used in the MDD-W questionnaire, sampling framework, survey timing, and data collection modality. In addition, using data from two non-inferiority studies in Ethiopia – which used either a weighed food record (WFR) or quantitative 24-hour recall (24-HR) as the reference method – we simulated the total magnitude of errors associated with non-quantitative open or list-based 24-HRs, and subsequently compared the pp differences in simulated food group consumption and MDD-W prevalence.

**Results:**

MDD-W estimates from the GWP were significantly higher than those from the DHS in five of nine country-year sets, one difference was non-significant, and three estimates could not be statistically compared due to lack of reporting on margins of error. The absolute difference between MDD-W estimates from the DHS and GWP were >|5| pp for all country-year sets (range: -17 to + 21 pp). There was poor agreement between the DHS and GWP questionnaires regarding the choice and number of sentinel foods used for each food group in the same country (range: 21 to 65%). In general, GWP data collection covered fewer months and questionnaires were enumerated in fewer languages than the DHS, but the number of sentinel foods used per food group was more standardized across countries. Simulations indicated that the magnitude of errors associated with pilot tested non-quantitative open and extensive list-based 24-HRs were marginally lower than sentinel list-based 24-HRs in Ethiopia.

**Conclusion:**

For global monitoring, standards must be defined for the preferred data collection instrument and survey platform for each healthy diet metric. This would facilitate cross-country comparability and help mitigate misinterpretations of change over time within countries and the selective reporting of national statistics. A harmonized methodology for developing, pilot testing, and continuously updating sentinel food lists is needed to further improve the accuracy of MDD-W questionnaires.

**Supplementary Information:**

The online version contains supplementary material available at 10.1186/s40795-025-01065-7.

## Introduction

Unhealthy diets are a leading cause of morbidity and mortality globally [1, 2]. While gradual progress has been made on collecting nationally representative quantitative dietary intake data, diets remain infrequently monitored on a large scale worldwide [3]. In response to the financial and human resource burden and lagged data dissemination associated with quantitative dietary assessment, low-burden brief data collection instruments have been developed – which are conventionally qualitative recalls of food groups consumption [4]. Nevertheless, there remains a lack of consensus on the measures and indicators that best capture the four priority sub-constructs of a healthy diet for monitoring purposes (i.e., diversity, nutrient adequacy, macronutrient balance, and moderation) [5]. Consequently, healthy diet indicators have been omitted in global monitoring frameworks, such as the Sustainable Development Goals and the World Health Assembly global nutrition targets. Due to the rapid pace of changes across food systems and the associated dietary transition, however, the importance of monitoring what people eat and drink across population groups and countries has never been more critical [6].

In 2023, Healthy Diets Monitoring Initiative (HDMI) – a partnership among FAO, UNICEF, and WHO – identified four healthy diet metrics for which routine data collection was deemed most promising at the global level [7]. Low-burden questionnaires for one priority metric, the MDD-W indicator [8], have already been included in two multi-country nationally representative data collection platforms, namely DHS (i.e., Phase 8 Woman’s Questionnaire) and GWP (i.e., Global Diet Quality Project), and many country-specific national and sub-national surveys. Nonetheless, the MDD-W questionnaires used in the DHS and GWP are known to differ in their disaggregation of MDD-W food groups, the total number of food groups included, and the number and examples of foods listed under each food group.

To track national level progress on healthy diets, which also informs global monitoring frameworks, timely, precise, and accurate dietary data are needed [9, 10]. Therefore, it is desirable that prevalence estimates of healthy diet indicators, such as MDD-W, for the same country collected in the same year, be consistent across various surveys to ensure robust monitoring at national level. Evidence for poor precision might hinder the combined use of data collection platforms and might indicate inaccurate data from one or multiple sources. In recognition of this concern, this study assessed the measurement agreement of food group consumption and MDD-W prevalence for available country-year sets, using meta-data from the DHS and GWP, and qualitatively examined the potential causes for disagreement.

However, without an objective reference method, the aforementioned data cannot determine which data collection platform yields the most accurate estimate of MDD-W. The non-quantitative list-based 24-HR method used for DHS and GWP has previously been shown to overestimate MDD-W prevalence among non-pregnant females, as compared to direct quantitative observations [11, 12] or quantitative 24-HRs across multiple countries [13]. Therefore, using data from two non-inferiority studies in Ethiopia, this study also aimed to compare the magnitudes of errors associated with each brief data collection instrument, by adjusting published estimates for the distinct reference measures used (i.e., accounting, where necessary, for respondent biases), and to compare the difference in simulated food group consumption and MDD-W prevalence.

## Methods

### MDD-W indicator

Achieving MDD-W is defined as a female consuming foods from at least five out of ten food groups over the previous 24 h. The ten predefined food groups are: (1) grains, white roots and tubers, and plantains; (2) pulses (beans, peas, and lentils); (3) nuts and seeds; (4) milk and milk products; (5) meat, poultry and fish; (6) eggs; (7) dark green, leafy vegetables; (8) vitamin A-rich fruits and vegetables; (9) other vegetables; and (10) other fruits. The proportion of females in the survey population who meet this cutoff is defined as the MDD-W prevalence [14–16].

### Data sources: comparing food group consumption and MDD-W prevalence from DHS and GWP conducted in the same countries

Meta-data on food group consumption and MDD-W prevalence and survey details were manually extracted from reports and websites described in detail below.

In 2019, a questionnaire on food group consumption was included in the DHS Phase 8 Woman’s Questionnaire. The data collected through the non-quantitative sentinel, or irregularly extensive, food list-based 24-HR allows for computation of the MDD-W indicator among (non-pregnant) females aged 15–49 years. Recent DHS adaptations of the MDD-W questionnaire start from the public goods shared by the Global Diet Quality Project. The DHS Program reviews the sentinel foods and makes changes based on suggestions from DHS key informants, before sharing them with the implementing agencies. These agencies themselves frequently offer feedback, which is subsequently circled back to the Global Diet Quality Project to determine if changes can be accommodated. DHS enumerators are trained to read each sentinel food listed to the respondent under the relevant question [17]. On 24 January 2024, at the time of data analysis, the DHS Program website indicated that food group consumption data were collected, and MDD-W point estimates were published in final survey reports, in nine countries [i.e., Burkina Faso [18], Cambodia [19], Kenya [20], Nepal [21], Nigeria [22], the Philippines [23], Sierra Leone [24], Tajikistan [25], and Tanzania [26]], while data collection or final survey report finalization was ongoing for 14 more countries.

In 2021–2023, a broader questionnaire on food group consumption, known as the Diet Quality Questionnaire (DQQ), was included in the GWP. The data collected through the non-quantitative sentinel food list-based 24-HR allows for the computation of multiple metrics, including the MDD-W indicator among females aged 15–49 years. The DQQ country-level adaptation protocol includes a desk review of relevant diet-related data and literature, iterative interviews with 5–10 key informant to identify the most commonly consumed (i.e., sentinel) foods and their vernacular names, identification of anomalous food items from regional-level comparisons of DQQs, and harmonization of terminology used [27]. Enumerators are instructed to read the DQQ exactly as written, without additional dialogue or probing. On 24 January 2024, the Global Diet Quality Project website indicated that MDD-W point estimates were available for 56 countries, including the nine aforementioned countries for which estimates are also available from DHS. Between 2023 and 2024, DQQ data collection was ongoing through the GWP for an additional 36 countries. On 19 June 2024, at the time of writing, disaggregated summary statistics for MDD-W were available for 85 countries on the DQQ Dataverse website [28].

### Data sources: partitioning misclassifications by brief MDD-W data collection instruments as compared to reference methods in Ethiopia

Meta-data, and available micro-data, on food group consumption and MDD-W prevalence were extracted from two published non-inferiority studies described in detail below.

Uyar et al. (2023) analysed data from 488 females (aged 15–49 years) collected between November and December 2019 in five regions of Ethiopia, namely, Amhara, Oromia, SNNPR, Tigray, and Addis Ababa [13]. MDD-W point estimates from the DQQ (7.4%; *n =* 969) were compared to estimates from quantitative 24-HRs enumerated on the same day (1.3%; *n =* 969). Food group misclassifications could therefore only be attributed to DQQ-related misestimations (e.g., too few or incorrect sentinel foods on the food list). Indeed, without an objective reference measure, such as WFRs, respondent biases, such as intrusions (i.e., respondent recalls consuming a food group, when a WFR objectively indicates no foods belonging to that food group were consumed) and omissions (i.e., respondent fails to recall consuming a food group, when a WFR objectively indicates foods belonging to that food group were consumed) of foods and drinks, cannot be identified due to the correlated errors of both recall-based methods [29].

Hanley-Cook et al. (2024) analysed data from 431 non-pregnant females (aged 15–49 years) collected between June and August 2019 in the Amhara Region of Ethiopia [12]. MDD-W prevalence from non-quantitative open (12%) and (extensive) food list-based 24-HRs (16%) were compared to WFRs enumerated on the day prior (8%). This objective reference measure allowed food group misclassifications to be partitioned into errors related to respondent biases (e.g., memory lapses, social desirability) or brief data collection instrument misestimations (e.g., incomplete food list). For most food groups, the predominant sources of overreporting (i.e., > 80% of cases) by both brief data collection instruments were intrusions [12].

### Statistical analysis

Data management, statistical analysis, and data visualizations were conducted in Stata version 16.1 [30]. Descriptive data are presented as frequencies and percentages, while absolute differences are presented in pp with 95% confidence intervals (CIs), whenever possible.

### Degree of concordance between DHS and GWP

We used the point estimates of MDD-W prevalence and consumption prevalence of the 10 underlying food groups published in the final DHS reports and on the Global Diet Quality Project website to visualize (i.e., range plots) and subsequently assess the absolute difference (i.e., precision) between the DHS and GWP for nine overlapping countries. To assess if absolute differences were statistically significant, we used the 95% CIs from the GWP^1^ and the available 95% CIs (i.e., ± 2 × standard error) published in final reports by DHS. If the absolute difference between the point estimates from the GWP and DHS was positive, we compared the magnitude of the difference between the lower limit of the GWP 95% CI against the upper limit of the DHS 95% CI.

The Global Diet Quality Project publishes consumption prevalence for “vitamin A-rich orange vegetables” and “vitamin A-rich fruits” separately, which does not allow for the calculation of national consumption estimates of the MDD-W indicator’s “vitamin A-rich fruits and vegetables” food group. Likewise, the Global Diet Quality Project publishes consumption prevalence for “citrus” and “other fruits” separately, which does not allow for the calculation of national consumption estimates of the MDD-W indicator’s “other fruits” food group as it strictly also includes citrus foods. Therefore, potential disagreement of consumption prevalence between the DHS and GWP could not be assessed for these two food groups. However, this has no effect on the discordance analyses for the other eight food groups or MDD-W prevalence.

In addition, in consultation with the HDMI, four broad survey characteristics were defined to qualitatively assess the potential disagreement between estimates from the DHS and GWP:

i. Food group questionnaire (e.g., choice and number of sentinel foods).
ii. Sampling framework and sample size (e.g., exclusion of regions).
iii. Timing of data collection (e.g., in a single or across multiple seasons).
iv. Data collection modality (e.g., face-to-face or phone-based interview, languages).

To quantitatively assess the agreement between the sentinel foods listed on the DHS questionnaire and DQQ, two nutrition experts on the study team standardized the food descriptors. For example, in Kenya, “jute mallow” or “mrenda” were both coded with a uniform descriptor “jute mallow,” with the aim to mitigate artificial disagreement based on terminology used. Subsequently, 2 × 2 contingency tables were used to compare the total number of sentinel foods on both questionnaires and the overlap of sentinel foods on the shorter questionnaire only (i.e., the share of foods on the shorter list that also appear on the longer list).

The rank correlation between the absolute difference in MDD-W prevalence and the percent agreement of sentinel foods was assessed using Spearman’s *ρ*.

### Accuracy of DQQ as compared to simulated WFR in Ethiopia

We used the published meta-data in Uyar et al. (2023) [13] to estimate the absolute discordance (i.e., accuracy) of the 10 MDD-W food group consumption prevalence from the DQQ in Ethiopia, when accounting for the proportion of error attributable to respondent biases identified for the list-based 24-HR by Hanley-Cook et al. (2024) (i.e., assuming this best approximates using WFRs as a reference measure) [12]. The relative inflation factor (RIF) attributable to respondent biases was computed as follows:

$$\:\frac{(a-b)}{(\left|a-b\right|+c)}$$ × 100

, where *a* is the number of individuals with intrusions for a specific food group, *b* is the number of individuals with omissions for the same food group, and *c* is the number of misestimations (i.e., food item on food list was consumed, but in < 15 g/day). Subsequently, the estimated pp difference in food group consumption prevalence between the DQQ and simulated WFR was computed as follows:

$\:\left(\frac{100}{100-\text{R}\text{I}\text{F}}\:\right)\:$× Δ quantitative 24-HR % – DQQ %

For food groups which, according to Hanley-Cook et al. (2024) [12], were not subject to misestimations but only respondent biases, the absolute inflation factor (AIF) was simply calculated as the difference between the proportion of individuals reporting intrusions minus and individuals reporting omissions during the (extensive) list-based 24-HR. Consequently, the estimated pp difference in the food group consumption prevalence between the DQQ and simulated WFR was computed as follows:

Δ quantitative 24-HR % – DQQ % + AIF.

As a comparative analysis, we assessed the impact of using only the AIF, rather than the RIF, on the difference in food group consumption prevalence between the DQQ and simulated WFR for food groups which were in fact subject to a degree of misestimation.

### Accuracy of open and (extensive) list-based 24-HR as compared to simulated quantitative 24-HR in Ethiopia

We used the published meta-data in Hanley-Cook et al. (2024) [12] to estimate the discordance of the 10 MDD-W food group consumption estimates from the open and (extensive) food list-based 24-HRs (henceforth referred to as brief instruments) in Ethiopia, when completely discounting the proportion of error attributable to respondent biases (i.e., assuming that a perfect correlation of errors between recall-based methods approximates using quantitative 24-HRs as the reference measure). The relative deflation factor (RDF) attributable to respondent biases was computed identically to the RIF. Subsequently, the estimated pp difference in food group consumption prevalence between the brief instrument and simulated quantitative 24-HR was computed as follows:

Δ WFR % – brief instrument % × $\:\left(\frac{100-\text{R}\text{D}\text{F}}{100}\:\right)$

Moreover, we also re-analysed the individual-level micro-data used in Hanley-Cook et al. (2024) [12] to estimate the discordance of the MDD-W prevalence from the brief instruments in Ethiopia, when completely discounting the proportion of error attributable to respondent biases. To this end, individuals with intrusions for a food group, were recoded as non-consumers and individuals with omissions for a food group were recoded as consumers. Subsequently, we re-calculated the MDD-W prevalence from the adjusted brief instruments and assessed the pp difference as compared to the WFR prevalence.

As a comparative analysis, we estimated the discordance of MDD-W prevalence for brief instruments in Ethiopia (i.e., analyses repeated for open and list-based recall), when accounting for the proportion of errors attributable to respondent biases to the WFR. In this case, individuals with intrusions for a food group were recoded as consumers and individuals with omissions for a food group were recoded as non-consumers.

## Results

### Degree of concordance between DHS and GWP

#### MDD-W and food group consumption prevalence

**Fig. 1:**
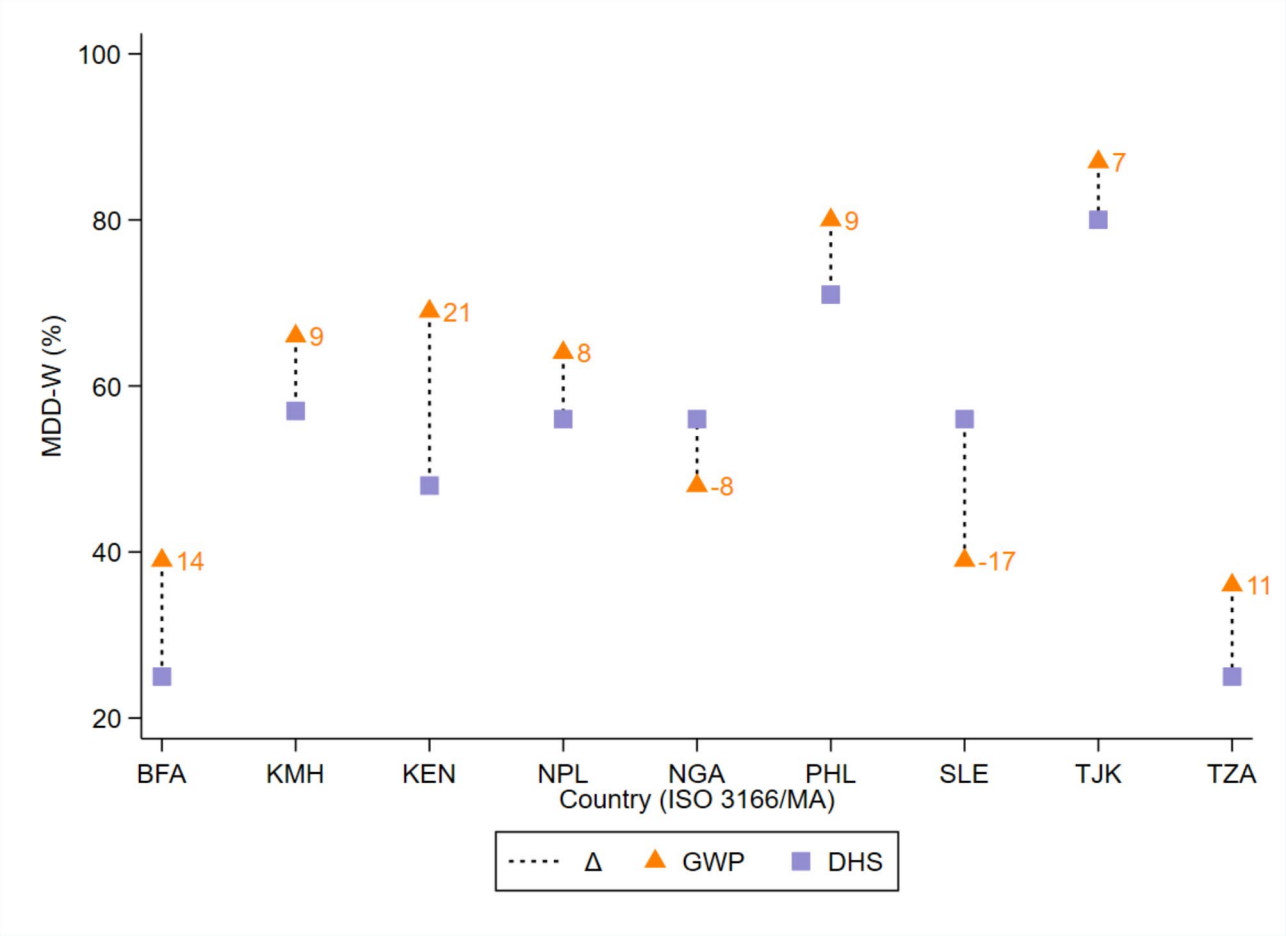
Difference between nationally representative Demographic and Healthy Survey (DHS) and Gallup World Poll (GWP) estimates of MDD-W prevalence, among females (15–49 years) in nine low- and middle-income countries. Y-axis ranges between a minimum of 20% and a maximum of 100%. Δ, absolute difference in percentage points between DHS and GWP; BFA, Burkina Faso; ISO, International Organization for Standardization; KEN, Republic of Kenya; KMH, Kingdom of Cambodia; NGA, Federal Republic of Nigeria; NPL, Nepal; PHL, Republic of the Philippines; SLE, Republic of Sierra Leone; TJK, Republic of Tajikistan; TZA, United Republic of Tanzania

**Fig. 2:**
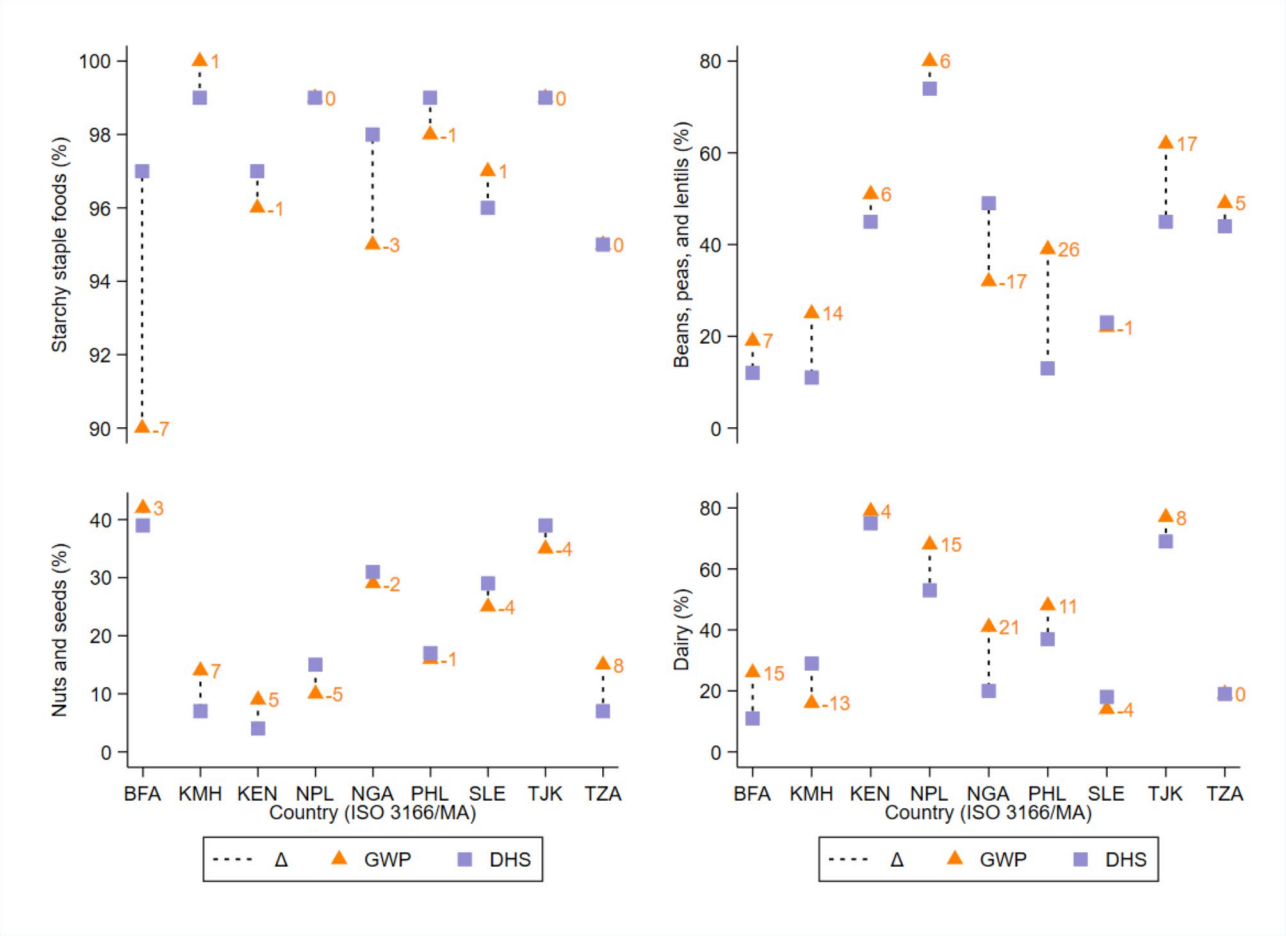
Difference between nationally representative Demographic and Healthy Survey (DHS) and Gallup World Poll (GWP) consumption estimates of “starchy staples,” “pulses,” “nuts and seeds,” and “dairy” food groups, among females (15–49 years) in nine low- and middle-income countries. Y-axis range differs for each food group. Δ, absolute difference in percentage points between DHS and GWP; BFA, Burkina Faso; ISO, International Organization for Standardization; KEN, Republic of Kenya; KMH, Kingdom of Cambodia; NGA, Federal Republic of Nigeria; NPL, Nepal; PHL, Republic of the Philippines; SLE, Republic of Sierra Leone; TJK, Republic of Tajikistan; TZA, United Republic of Tanzania

**Fig. 3:**
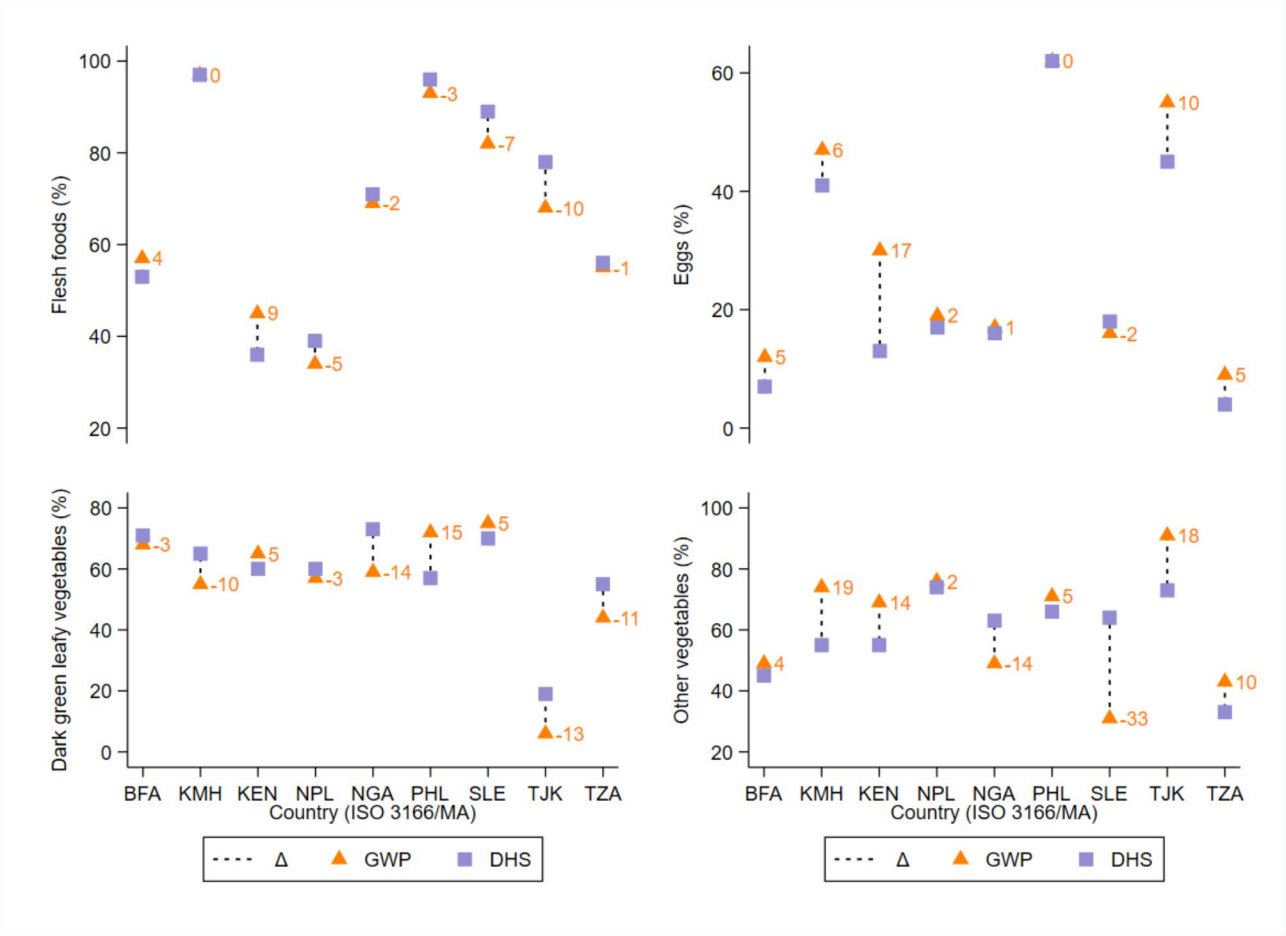
Difference between nationally representative Demographic and Healthy Survey (DHS) and Gallup World Poll (GWP) consumption estimates of “flesh foods,” “eggs,” “dark green leafy vegetables,” and “other vegetables” food groups, among females (15-49 years) in nine low- and middle-income countries. Y-axis range differs for each food group. Δ, absolute difference in percentage points between DHS and GWP; BFA, Burkina Faso; ISO, International Organization for Standardization; KEN, Republic of Kenya; KMH, Kingdom of Cambodia; NGA, Federal Republic of Nigeria; NPL, Nepal; PHL, Republic of the Philippines; SLE, Republic of Sierra Leone; TJK, Republic of Tajikistan; TZA, United Republic of Tanzania

The MDD-W point estimates from the DHS and GWP differed by >|5| pp for all nine country-year sets [mean:|11.6| pp; range: -17 pp in Sierra Leone and + 21 pp (95% CI: 13, 28) in Kenya]. Furthermore, the MDD-W estimates from the GWP were significantly higher than those from the DHS in five of nine country-year sets (mean: 12.8 pp) (Fig. 1; Table 1). While the GWP point estimate in Nepal was 8 pp higher than the DHS in Nepal, the difference was not statistically different (95% CI Δ: -1, 17 pp). No variability estimates were published in the DHS reports for three countries. Nevertheless, under the conservative assumption of a margin of error of ± 2 pp, the MDD-W estimates from the DHS in Nigeria and Sierra Leone would be significantly higher than those from the GWP. The opposite conclusion would be reached for Tajikistan, noting however that the DHS and GWP were collected four years apart. The food groups most prone to a higher consumption prevalence through the GWP were “beans, peas, and lentils” (i.e., seven countries), “dairy” (i.e., six countries), and “other vegetables” (i.e., seven countries) (Figs. 2–3).

**Table 1:** Description of gallup world poll (GWP) and demographic healthy surveys (DHS) for nine low- and middle-income countries for which minimum dietary diversity for women estimates are available

Country	BFA		KMH		KEN		NPL		NGA		PHL		SLE		TJK		TZA	
Survey	GWP	DHS	GWP	DHS	GWP	DHS	GWP	DHS	GWP	DHS	GWP	DHS	GWP	DHS	GWP	DHS	GWP	DHS
**Time**,** mm/yyyy**	08/2021–09/2021	07/2021–11/2021	08/2021–10/2021	09/2021–02/2022	06/2021–07/2021	02/2022–07/2022	09/2021–11/2021	01/2022–06/2022	07/2021–08/2021	08/2018–12/2021	08/2021–10/2021	05/2022–06/2022	06/2021–07/2021	05/2019–08/2019	08/2021–10/2021	08/2017–11/2017	08/2021	02/2022–07/2022
**Sample size**, ***n***^**1**^	419	17,659	420	19,496	453	32,156	416	14,845	436	41,821	530	27,821	595	15,574	524	10,718	495	15,254
**MDD-W** **(95% CI)**,** %**^**2**^	39 (32, 46)	25 (24, 27)	66 (59, 73)	57 (56, 59)	69 (63, 75)	48 (47, 50)	64 (57, 71)	56 (54, 58)	48 (42, 54)	56	80 (75, 85)	71 (69, 72)	39 (34, 44)	56	87 (83, 90)	80	36 (30, 42)	25 (24, 27)
**Margin of error**,** %**^**3**^	–7.0, + 7.2	± 1.4	–6.9, + 6.6	± 1.4	–5.9, + 5.6	± 1.2	–6.9, + 6.6	± 1.8	–5.9, + 5.9	NA	–5.2, + 4.8	± 1.4	–4.8, + 4.9	NA	–4.0, + 3.5	NA	–5.6, + 5.8	± 1.6
**Mode of interview**	F2F	F2F	F2F	F2F	F2F	F2F	F2F	F2F	F2F	F2F	Mobile telephone	F2F	F2F	F2F	F2F	F2F	F2F	F2F
**Languages of questionnaire**	Dioula, French, Fulfide, Moore	Dioula, French, Fulfide, Moore, Gourmanché, Dagara	Khmer	Khmer, English	English, Swahili	English, Swahili, Borana, Embu, Kalenjin, Kamba, Kikuyu, Kisii, Luhya, Maragoli, Luo, Maasai, Meru, Mijikenda, Pokot, Somali, Turkana	Nepali	Nepali, English, Maithili, Bhojpuri	English, Hausa, Yoruba, Igbo, Pidgin English	English, Hausa, Yoruba, Igbo	Filipino, Ilokano, Cebuano, Bikolano, Waray	English, Ilokano, Cebuano Bikolano, Waray, Tagalog, Hiligaynon	English, Krio, Mende	English, Krio, Mende, Temne, Limba	Tajik	Tajik English, Tajik, Russian	Swahili	Swahili, English, Kikongo

^1^Females aged 15–49 years only. BFA, Burkina Faso; CI, confidence interval; F2F, face-to-face; KEN, Republic of Kenya; KMH, Kingdom of Cambodia; NA, not available; NGA, Federal Republic of Nigeria; NPL, Nepal; PHL, Republic of the Philippines; pp, percentage points; SLE, Republic of Sierra Leone; TJK, Republic of Tajikistan; TZA, United Republic of Tanzania

^2^For the GWP, the 95% CIs were calculated using the variance-stabilizing transformation for the binomial distribution, the arcsine square root, and then back-transforming the 95% CIs to the probability scale (personal communication with Drs. Anna Herforth and Ty Beal)

^3^The symmetrical margins of error around the MDD-W prevalence are published in the technical annexes of final DHS reports

### Food group questionnaires

Overall, the agreement between the (sentinel) foods on the DHS questionnaire and DQQ was relatively poor, ranging from 21% of total foods in Sierra Leone up to 65% in the Philippines. Nonetheless, the overlap of food items included only on the shorter of the two questionnaires, in other words considering the shorter list as the denominator, was > 75% in six countries, ranging between 48% in Tajikistan and 93% in Nepal (Table 2). The absolute difference in MDD-W prevalence was not significantly rank correlated with the percent agreement of sentinel foods (*ρ*=-0.12; *P* = 0.73).

**Table 2:** Agreement between food item examples listed under the 10 Minimum Dietary Diversity for Women (MDD-W) food groups from the Gallup World Poll (GWP) and Demographic Healthy Surveys (DHS)^1^

	DHS		Agreement statistics	
	Included	Excluded	% Total agreement of food items^2^	% Agreement of food items on shortest questionnaire
	**BFA**			
**GWP**				
Included	62	34	54.9	78.5
Excluded	17	-		
	**KMH**			
**GWP**				
Included	62	44	51.2	80.5
Excluded	15	-		
	**KEN**			
**GWP**				
Included	75	33	62.0	85.2
Excluded	13	-		
	**NPL**			
**GWP**				
Included	63	34	61.8	92.6
Excluded	5	-		
	**NGA**			
**GWP**				
Included	55	52	33.7	51.4
Excluded	81	-		
	**PHL**			
**GWP**				
Included	72	27	64.9	85.7
Excluded	12	-		
	**SLE**			
**GWP**				
Included	52	38	21.0	57.8
Excluded	158	-		
	**TJK**			
**GWP**				
Included	41	49	30.6	48.2
Excluded	44	-		
	**TZA**			
**GWP**				
Included	62	38	51.7	75.6
Excluded	20	-		

^1^Values are frequencies. BFA, Burkina Faso; KEN, Republic of Kenya; KMH, Kingdom of Cambodia; NGA, Federal Republic of Nigeria; NPL, Nepal; PHL, Republic of the Philippines; SLE, Republic of Sierra Leone; TJK, Republic of Tajikistan; TZA, United Republic of Tanzania

^2^Food items included in both the DHS and GWP ÷ (food item included in both the DHS and GWP + food items included in the DHS only + food items included in the GWP). To illustrate, the total agreement of food items between the DHS and GWP for BFA is: 62 ÷ (62 + 17 + 34) = 54.9%

^3^Food items included in both the DHS and GWP ÷ (food item included in both the DHS and GWP + food items included in the shortest survey only). To illustrate, in BFA, the DHS included 79 food items, while the GWP included 96 food items. Hence, the agreement of food items on the shorter DHS questionnaire also captured by the longer GWP for BFA is: 62 ÷ (62 + 17) = 80.5%

In seven countries, for the MDD-W relevant questions, the DQQ listed more sentinel food items than the DHS questionnaire. Furthermore, for all country-year sets, the DQQ was composed of more individual MDD-W relevant questions due to a greater disaggregation of the 10 food groups, leading to fewer sentinel foods per question. The DHS Sierra Leone questionnaire incorrectly included ice-cream and condensed milk as food item examples for the “milk and milk products” food group. The food groups with the greatest number of (sentinel foods) in the GWP and DHS were “starchy staples” [mean (SD): 17 [2] vs. 15 [4]], “flesh foods” [mean (SD): 20 [2] vs. 24 [17]], and “other fruits” [mean (SD): 11 [9]]. A maximum of 35 and 67 foods were listed under the “dark green leafy vegetables” in the DHS Nigeria and “flesh foods” in the DHS Sierra Leone.

To calculate MDD-W, data from either 22 (e.g., Nepal) or 23 (e.g., Burkina Faso) questions on the DQQ were required. In contrast, the available DHS included between 14 (e.g., Sierra Leone) and 16 relevant questions (e.g., Cambodia) for MDD-W computation.

### Sampling framework and sample size

The DHS samples females and, frequently, males aged 15–49 years and their children aged 0–59 months from the entire non-institutionalized civilian population. At present, MDD-W data are collected among females only. Data are collected through face-to-face, computer-assisted personal interviews. DHS usually follows a stratified two-stage sampling design. The first stage involves selection of clusters consisting of enumeration areas, generally from the most recent census frame. The enumeration areas are selected with a probability proportional to their size within each sampling stratum (e.g., region, urban and rural areas within regions). In the second stage, ~ 25–30 households are selected systematically from each cluster. A complete household listing is conducted in each of the selected clusters and acts as the sampling frame.

Due to the non-proportional allocation of households to the different regions and sampling stratums, and the differences in response rates, sampling weights are required. Sampling weights are based on sampling probabilities separately for each sampling stage and for each cluster. For households, the sampling weight is the inverse of its household selection probability multiplied by the inverse of the household response rate in the stratum. Subsequently, for females, the individual survey weight is the household weight multiplied by the inverse of the individual response rate for females in the stratum.

The GWP samples individuals aged 15 years and over from the entire non-institutionalized civilian population. Most data in low- and middle-income countries are collected through ~ 1 h face-to-face, computer-assisted personal interviews. The GWP’s clustered sampling method is as follows. Primary sampling units (i.e., ~ 125 clusters of ~ 8 households) are stratified by population size (e.g., cities, towns, and villages) or geography (e.g., region) and clustering is achieved through one or more stages of sampling. Where population information is available (e.g., census listings), household sample selection is based on probabilities proportional to population size, otherwise simple random sampling is used. Random route procedures are used to select sampled households. Random respondent selection is achieved by using the Kish grid method.

The GWP constructs base sampling weights to account for oversampling and household size (i.e., to adjust for the probability of selection). Thereafter, post-stratification weights are constructed. Population statistics are used to weight the data by gender, age, and, where reliable data are available, education, or socioeconomic status.

In countries where telephone coverage represents at least 80% of the population (e.g., the Philippines) or is the customary survey methodology, ~ 30-minute telephone surveys are used which are based on either random-digit-dial or a nationally representative list of phone numbers. Random respondent selection is achieved by using either the latest birthday or Kish grid method. Typically, the design is not stratified but otherwise the other processes and procedures follow those used in the face-to-face design.

For the countries included in this study, the sample sizes from the GWP ranged between 416 females in Nepal and 595 in Sierra Leone, whereas the sample sizes from the DHS ranged between 10,718 females in Tajikistan and 41,821 in Nigeria. The much smaller sample sizes from the GWP are reflected in the larger variability around the MDD-W point estimates (Table 1).

### Exclusion areas

The DHS Kenya excluded one cluster in Mandera County due to insecurity, from a total of 1,692 clusters across the country. Mandera County as a whole is home to ~ 2% of the Kenyan population, and therefore one cluster within it represents an even smaller proportion of the total population.

The GWP in Burkina Faso excluded some communities in the East and Sahel regions for security reasons, representing 4% of the population. The DHS Burkina Faso report indicated that the MDD-W prevalence was 12.5% (*n* = 948; 95% CI: 7.9–17.1) and 24.6% (*n* = 618; 95% CI: 20.4–28.8) in the East and Sahel regions, respectively [national estimate: 25.1% (23.8–26.5)].

Likewise, the GWP in Nigeria excluded the states of Adamawa, Borno, and Yobe for safety and security reasons, representing 7% of the population. The DHS Nigeria report indicated that the MDD-W prevalence in these states were 41.9% (*n* = 903), 38.4% (*n* = 1,469), and 66.7% (*n* = 1,327), respectively [national estimate: 56%].

Furthermore, the GWP in Cambodia excluded the Koh Kong, Stueng Treng, Otdar Meanchey, and Kep provinces, representing approximately 3% of the population. The DHS Cambodia report indicated that the MDD-W prevalence was 48.8% (*n* = 681; 95% CI: 42.1–57.4) in the Koh Kong province, 62.0% (*n* = 809; 95% CI: 57.5–66.5) in the Stueng Treng province, 51.8% (*n* = 712; 95% CI: 44.5–59.1) in the Otdar Meanchey province, and 67.8% (*n* = 746; 95% CI: 63.7–72.0) in the Kep province (national MDD-W estimate: 57.3%; 95% CI: 55.9–58.7).

### Timing of data collection and modality

For most countries, the DHS data collection period covered ~ 5 months, while the GWP covered ~ 2 months, with the notable exception of the DHS Philippines which covered less than two months. The most noticeable differences in the timing of DHS and GWP data collection efforts were in Cambodia (i.e., dry vs. wet season), Kenya (summer dry and longer wet vs. winter dry season), Nepal (winter and spring vs. early autumn), and Tanzania (long rainy vs. dry season) (Table 1).

Furthermore, with the exception of the GWP in the Philippines (i.e., mobile telephone-based interview), all MDD-W questionnaires were enumerated face-to-face. In eight countries, the DHS questionnaire was translated and could be enumerated in more languages (range: 2–17 languages) than the DQQ (range: 1–5 languages) (Table 1).

### Accuracy of DQQ as compared to simulated WFR in Ethiopia^2^

For the “nuts and seeds,” “flesh foods,” and “other fruits” food groups, the largest proportion of total error, using WFR as the reference, was attributable to respondent biases. Nonetheless, due to a larger number of misestimations (i.e., using quantitative 24-HR as the reference), our simulations – which accounted for respondent biases – indicated that the DQQ’s consumption prevalence for the “dairy,” “dark green leafy vegetables,” “vitamin A-rich fruits and vegetables,” and “other fruits” food groups were the least accurate (i.e., > 5 pp difference). Our comparative analyses confirmed the main findings (Table 3).

**Table 3:** Estimated discordance of the 10 Minimum Dietary Diversity for Women (MDD-W) food group consumption prevalence from the Diet Quality Questionnaire (DQQ) in Ethiopia (*n* = 969), without and with accounting for the estimated proportion of errors attributable to respondent biases

Food group	24-HR %^1^	DQQ %^1^	Percentage point difference between 24-HR and DQQ^1^	Relative inflation factor attributable to respondent biases^3^	Estimated percentage point difference between DQQ and simulated WFR^8^	Percentage point difference attributable to respondent biases^9^	Estimated percentage point difference between DQQ and simulated WFR^10^
1. Starchy staple foods	99.8	98.3	-1.5	0	**-1.5**	0	-1.5
2. Beans, peas, and lentils	57.8	61.1	3.3	4.7	**3.5**	0.5	3.8
3. Nuts and seeds	0.9	1.0	0.1	94.1	**1.7**	3.7	3.8
4. Dairy	11.2	13.3	2.1	NA^4^	**5.1** ^**4**^	3.0	5.1
5. Flesh foods	8.3	8.8	0.5	87.5	4.0	1.6	**2.1**
6. Eggs	2.6	3.0	0.4	NA^5^	1.3^5^	0.7	**1.1**
7. Dark green leafy vegetables	32.0	32.5	0.5	NA^6^	**5.6** ^**6**^	5.1	5.6
8. Vitamin A-rich fruits and vegetables	8.3	12.1	3.8	70.6	12.9	2.8	**6.6**
9. Other vegetables	NA^2^	NA^2^	NA^2^	NA^7^	**-1.9** ^7^	-1.9	NA^2^
10. Other fruits	3.7	6.9	3.2	82.8	18.6	5.7	**8.9**

^1^Estimates published by Uyar et al. (2023) [13]. 24-HR, 24-hour recall; NA, not applicable; WFR, weighed food record

^2^Could not be compared because DQQ contained an incorrect food item “green beans,” which respondents understood as legumes that are green [13]

^3^Overreporting estimates from (extensive) list-based 24-HRs as compared to weighed food records are published in Table 4 of Hanley-Cook et al. (2024) [12], while underreporting estimates are available in Supplementary Table 1 of the current manuscript. The error inflation factor attributable to respondent biases is computed as follows: (*a* – *b*) ÷ (*|a – b|* + *c*), where *a* is the number of individuals reporting consuming a food group when the WFR objectively indicated no food items belonging to the respective food group were consumed, *b* is the number of individuals not reporting consuming a food group when the WFR objectively indicated food items belonging to the respective food group were consumed, and *c* is the number of misestimations. To illustrate for the beans, peas, and lentils food group: (11–9) ÷ (11–9 + 41) = 4.7%

^4^3.0% of individuals reported consuming dairy when the WFR objectively indicated no food items belonging to the respective food group were consumed. Furthermore, there were no misestimations recorded. Consequently, the absolute inflation factor attributable to respondent biases was estimated as 3.0% points

^5^0.9% of individuals reported consuming eggs when the WFR objectively indicated no food items belonging to the respective food group were consumed, while 0.2% of individuals did not report consuming eggs when the WFR objectively indicated food items belonging to the respective food group were consumed. Furthermore, there were no misestimations recorded. Consequently, the absolute inflation factor attributable to respondent biases was estimated as 0.7% points

^6^5.8% of individuals reported consuming dark green leafy vegetables when the WFR objectively indicated no food items belonging to the respective food group were consumed, while 0.7% of individuals did not report consuming dark green leafy vegetables when the WFR objectively indicated food items belonging to the respective food group were consumed. Furthermore, there were no misestimations recorded. Consequently, the absolute inflation factor attributable to respondent biases was estimated as 5.1% points

^7^1.9% of individuals did not report consuming other vegetables when the WFR objectively indicated food items belonging to the respective food group were consumed, while misestimations were recorded for 4.4% of individuals. Consequently, the relative inflation factor attributable to respondent biases is -29.6%. In other words, the percentage point difference between 24-HR and DQQ is theoretically reduced by underreporting, in part, cancelling out misestimations. However, as the consumption prevalence of other vegetables could not be estimated, the absolute inflation factor attributable to respondent biases was estimated as -1.9% points

^8^100 ÷ (100 – value in column 5) × value in column 4

^9^Proportion of overreporters – proportion of underreporters. To illustrate for the beans, peas, and lentils food group: (11 ÷ 431) – (9 ÷ 431) = 0.5% points

^10^Column 4 + column 7

### Accuracy of open and (extensive) list-based 24-HR as compared to simulated quantitative 24-HR in Ethiopia

When discounting the proportion of total error attributable to respondent biases, using WFR as the reference, our simulations indicated that both the open and list-based 24-HRs were highly accurate for eight food groups (<|1.5| pp difference), while the consumption prevalence for the “beans, peas, and lentils” food group were the least accurate (i.e., > 9 pp difference) (Tables 4 and 5, Supplemental Table 1).

**Table 4:** Estimated discordance of the 10 Minimum Dietary Diversity for Women (MDD-W) food group consumption prevalence from the non-quantitative list-based 24-HR in Ethiopia (*n* = 431), with and without discounting the proportion of errors attributable to respondent biases

Food group	WFR %^1^	List-based 24-HR %^1^	Percentage point difference between WFR and list-based 24-HR	Relative deflation factor attributable to respondent biases^2^	Estimated percentage point difference between list-based 24-HR and simulated quantitative 24-HR^4^
1. Starchy staple foods	100	100	0	0	0
2. Beans, peas and lentils	80.3	90.3	10.0	4.7	9.5
3. Nuts and seeds	0.7	4.6	3.9	94.1	0.2
4. Dairy	4.2	7.2	3.0	100^3^	0
5. Flesh foods	8.8	10.7	1.9	87.5	0.2
6. Eggs	5.8	6.6	0.8	100^3^	0
7. Dark green leafy vegetables	6.7	11.8	5.1	100^3^	0
8. Vitamin A-rich fruits and vegetables	11.6	15.1	3.5	70.6	1.0
9. Other vegetables	94.0	96.5	2.5	-29.6	3.2
10. Other fruits	4.4	10.9	6.5	82.8	1.1

^1^Estimates published by Hanley-Cook et al. (2024) [12]. 24-HR, 24-hour recall; WFR, weighed food record

^2^Overreporting estimates from (extensive) list-based 24-HRs as compared to weighed food records are published in Table 5 of Hanley-Cook et al. (2024) [12], while underreporting estimates are available in Supplementary Table 1 of the current manuscript. The relative deflation factor attributable to respondent biases is computed as follows: (*a* – *b*) ÷ (|*a* – *b|* + *c*), where *a* is the number of individuals reporting consuming a food group when the WFR objectively indicated no food items belonging to the respective food group were consumed, *b* is the number of individuals not reporting consuming a food group when the WFR objectively indicated food items belonging to the respective food group were consumed, and *c* is the number of misestimations. To illustrate for the beans, peas, and lentils food group: (11–9) ÷ (11–9 + 41) = 4.7%

^3^No misestimations recorded

^4^Value in column 4 × [(100 – value in column 5) ÷ 100]

**Table 5:** Estimated discordance of the 10 Minimum Dietary Diversity for Women (MDD-W) food group consumption prevalence from the non-quantitative open 24-HR in Ethiopia (*n* = 431), with and without discounting the estimated proportion of errors attributable to respondent biases

Food group	WFR %^1^	Open 24-HR %^1^	Percentage point difference between WFR and open 24-HR	Relative deflation factor attributable to respondent biases^2^	Estimated percentage point difference between open 24-HR and simulated quantitative 24-HR^4^
1. Starchy staple foods	100	100	0	0	0
2. Beans, peas and lentils	80.3	89.8	9.5	4.9	9.0
3. Nuts and seeds	0.7	3.7	3.0	100^3^	0
4. Dairy	4.2	7.2	3.0	100^3^	0
5. Flesh foods	8.8	10.7	1.9	87.5	0.2
6. Eggs	5.8	6.0	0.2	100^3^	0
7. Dark green leafy vegetables	6.7	8.7	2.0	100^3^	0
8. Vitamin A-rich fruits and vegetables	11.6	11.1	-0.5	-66.7	-0.8
9. Other vegetables	94.0	97.2	3.2	-25.0	4.0
10. Other fruits	4.4	6.5	2.1	55.6	0.9

^1^Estimates published by Hanley-Cook et al. (2024) [12]. 24-HR, 24-hour recall; WFR, weighed food record

^2^Overreporting estimates from open 24-HRs as compared to weighed food records are published in Table 5 of Hanley-Cook et al. (2024) [12], while underreporting estimates are available in Supplementary Table 1 of the current manuscript. The relative deflation factor attributable to respondent biases is computed as follows: (*a* – *b*) ÷ (|*a* – *b|* + *c*), where *a* is the number of individuals reporting consuming a food group when the WFR objectively indicated no food items belonging to the respective food group were consumed, *b* is the number of individuals not reporting consuming a food group when the WFR objectively indicated food items belonging to the respective food group were consumed, and *c* is the number of misestimations. To illustrate for the beans, peas, and lentils food group: (9–7) ÷ (9–7 + 39) = 4.9%

^3^No misestimations recorded

^4^Value in column 4 × [(100 – value in column 5) ÷ 100]

Adjusting the individual-level food group consumption data from the brief instruments (i.e., discounting errors due to respondent biases in the micro-data) showed that the open and list-based 24-HRs estimated MDD-W prevalence to within ~ 1 pp of the reference method (Table 6). Our comparative analyses, adjusting the individual-level food group consumption data from the WFR (i.e., accounting errors due to respondent biases in the micro-data) indicated that the brief instruments estimated MDD-W prevalence to within < 3 pp of the reference method (Supplemental Table 2).

**Table 6:** Estimated discordance of Minimum Dietary Diversity for Women (MDD-W) prevalence from non-quantitative open and list-based 24-HRs in Ethiopia, when discounting the proportion of errors attributable to respondent biases from brief data collection instruments

Indicator	24-HR % (*n* = 969)^1^	DQQ % (*n* = 969)^1^	Percentage point difference between 24-HR and DQQ^1^	WFR % (*n* = 431)^2^	Adjusted open 24-HR % (*n* = 431)^3^	Percentage point difference between WFR and adjusted open 24-HR	Adjusted list-based 24-HR % (*n* = 431)^3^	Percentage point difference between WFR and adjusted list-based 24-HR
MDD-W	1.3	7.4	6.1	7.7 (5.5–10.6)	8.6 (6.3–11.6)	0.9 (0.8-1.0)	8.8 (6.5–11.9)	1.1 (1.0-1.3)

^1^Point estimates published by Uyar et al. (2023) [13]. 24-HR, 24-hour recall; NA, not applicable; WFR, weighed food record

^2^Point estimates published by Hanley-Cook et al. (2024) [12], while the 95% confidence intervals were calculated by the authors

^3^Adjusted by recoding brief instruments micro-data of individuals that reported consuming a food group, when the WFR objectively indicated no food items belonging to the respective food group were consumed, as non-consumers and individuals that reported not consuming a food group, when the WFR objectively indicated food items belonging to the respective food group were consumed, as consumers

## Discussion

MDD-W estimates from the GWP were significantly higher than those from the DHS in five of nine country-year sets, while point estimates were likely to be statistically higher for an additional country but lower for two other countries. The variability around the MDD-W point estimates from the GWP was ~ 3.5 larger than those from the DHS in each country. The lack of precision between the DHS and GWP poses problems for reconciling national estimates of MDD-W across and within countries, when collected using different data collection platforms. Moreover, a calibration of prevalence estimates does not seem viable as the direction and the absolute or relative magnitude of the observed measurement disagreement was not systematic across countries.

Overall, sparse MDD-W estimates from other national surveys are more closely aligned with DHS than GWP. To illustrate, the 2023 Burkina Faso National Nutrition Survey reported an MDD-W prevalence of 18% (95% CI: 15–20) [31], as compared to 25% (95% CI: 24, 27) from the 2021 DHS [18]. Furthermore, the 2016 Nepal National Micronutrient Status Survey indicated an MDD-W prevalence of 49% (95% CI: 46–52) [32], in comparison with 50% in the 2016 DHS [33]. Moreover, the 2021 Sierra Leone National Nutrition Survey published an MDD-W prevalence of 74% (95% CI: 72–78) [34], which is 18 and 35 pp higher than the 2019 DHS [24] and 2021 GWP, respectively. Lastly, the 2016 National Nutrition Survey in Tajikistan estimated MDD-W prevalence at 81% [35], which is within 1 pp of the 2017 DHS [25].

We identified that there was poor agreement between the DHS and GWP questionnaires regarding the choice and number of sentinel foods used for each MDD-W food group. However, DHS has recently partnered with the Global Diet Quality Project, which has developed a global database of over 120 DQQs with relevant questions, to fine-tune their own MDD-W questionnaires moving forward [36]. Therefore, a harmonized methodology for developing and updating sentinel food lists, both within and across countries, and a standardized questionnaire used across national data collection platforms is likely to enhance the comparability and accuracy of MDD-W estimates moving forward. For most country-year sets, GWP data collection covered fewer months and questionnaires were enumerated in fewer languages than the DHS. The number of foods listed per question was more standardized in the GWP (i.e., no more than ~ 7 sentinel foods) but for seven country-year sets, the total number of foods enumerated was greater than the DHS due to the larger number of questions and greater disaggregation of MDD-W food groups.

The observed variability in the length of data collection and the seasons covered are likely to have contributed to the differences between the MDD-W estimates from the DHS and GWP. A longitudinal study among 503 mothers in Uganda showed significant fluctuations in the MDD-W prevalence across six-month intervals between November 2014 and May 2016 (i.e., females were more likely to achieve MDD-W shortly after the rainy season) [37]. Similarly, a longitudinal study among 167 mothers in Timor-Leste found significant variability in MDD-W prevalence across three to five month intervals between 2017 and 2018 [38]. Moreover, two longitudinal studies among 200 females of reproductive age in Malawi and Zambia showed that across eleven 24-HRs between September 2017 and May 2018 there were significant seasonal fluctuations in the percentage of females achieving MDD-W, peaking in the harvest season and lowest in the lean season [39]. Likewise, a longitudinal study among over 350 females of reproductive age in Ethiopia indicated that between February 2017 and August 2018 the MDD-W prevalence significantly differed across seasons (i.e., lowest during the Orthodox fasting and irrigation season) [40]. In addition, high-frequency longitudinal data from over 1,700 pregnant females in Burkina Faso showed that the MDD-W prevalence was responsive to seasonal variations, with peaks at the end of the dry season (i.e., April or May) and troughs in the rainy season (i.e., August) [41]. Lastly, a four-year longitudinal analysis of 2,701 mothers enrolled in a cluster-randomized controlled trial in Bangladesh indicated that MDD-W prevalence was prone to considerable seasonal fluctuations and was significantly higher in the pre- and early-monsoon months (May-July) [42]. These studies highlight that the consistent timing of data collection is critical for repeated surveys, such as DHS every ~ 5 years, even when using the same brief instrument, to ensure nonbiased inferences of change and trends in dietary diversity over time. Hence, various national surveys should also aim to align the months across which they collect dietary intake data – most conservative for MDD-W being the lean season – to improve the compatibility and comparability of estimates across platforms and countries. To illustrate, the GWP was implemented between August and September in Burkina Faso, which coincides with the lowest point of MDD-W prevalence, while the DHS was conducted between July and November, a period when MDD-W prevalence is likely to be substantially higher [41]. Therefore, the observed disagreement between the two data collection platforms might in fact be an underestimate for some countries. Neither the DHS or GWP publish data which allow an assessment of the distribution of dietary data collection across weekdays.

In addition, the narrower range of local languages in which data were collected through the GWP might have increased respondent burden and differential response bias among certain subgroups of the female population, as compared to DHS [43]. In DHS Jordan, enumerators have still reported the need to translate food or drinks items into local or slang names on the spot [17]. This finding reinforces the added-value of developing and adapting food lists with local experts, selecting local enumerators, and high-quality training and continuous feedback on the MDD-W questionnaire. Furthermore, the variable placement of the MDD-W questionnaire (e.g., near the beginning or the end of the survey) might be another cause for the differences between the DHS and GWP. To illustrate, a study that randomized the placement of the MDD-W questionnaire in a multi-topic phone-based survey in Ethiopia reported that delaying the questioning of food group consumption by 15 min led to a decrease in the 10-point food group diversity score [mean (SE) difference: 0.3 food groups (0.1)], although no difference in the MDD-W prevalence was observed [44]. In the DHS, the MDD-W questionnaire has been integrated into the “Child Health and Nutrition” section, which is the 6th of 12–16 sections of the Woman’s Questionnaire for the available country-year sets. To our knowledge, national GWP questionnaires, including the placement of the tailored DQQs, are not available as a global public good. Therefore, no comparisons could be made between the DHS and GWP for this survey characteristic.

Regarding data collection modality, the difference in MDD-W estimates from the Philippines is unlikely to be attributable to the mobile phone-based interview used in the GWP. A test-retest study comparing phone-based against face-to-face interviews among females of reproductive age in Kenya, with an interval of approximately one-week, indicated that the data collection modality had no effect on the MDD-W prevalence [45]. Moreover, it is improbable that the exclusion of specific regions in the GWP contributed substantially to the higher MDD-W estimates. With the exceptions of the East region in Burkina Faso and the Kep province in Cambodia, the DHS reports indicated that the MDD-W estimates from the excluded regions were comparable to the national prevalence.

These findings comparing DHS and GWP, however, fail to elicit which data collection platform provided the most accurate MDD-W estimate in each country-year set. For that purpose, (nationally-representative) dietary intake data would have to be collected among the same individuals using the DHS questionnaire, the DQQ, and a reference method of dietary intake, such as WFR on the day prior or quantitative 24-HR recall on the same day. These data would allow an assessment of the discordance of MDD-W estimates obtained through the DHS and GWP, as compared to a more objective MDD-W estimate or an examination of the predictive performance of these MDD-W estimates against reference metrics of nutrient adequacy (e.g., mean adequacy ratio of 11 micronutrients).

Our simulations indicated that the magnitude of errors associated with pilot tested non-quantitative open and (extensive) list-based 24-HRs were lower than a sentinel list-based 24-HRs in Ethiopia. However, when using the lenient non-inferiority criterion of ± 10% point from the reference method set by Uyar et al. (2023) [13], all brief instruments accurately estimated MDD-W prevalence as compared to (simulated) quantitative 24-HRs. These exploratory findings should not be extrapolated to other settings or population groups, as dietary patterns in Ethiopia are known to be more homogeneous and less diverse than in other countries [11, 13] and food group diversity and MDD-W prevalence has been shown to vary substantially across sexes in the same country [16]. Furthermore, for robust global monitoring of healthy diets, brief instruments must achieve stricter non-inferiority criteria. To this end, sentinel food lists might be optimized by relying more on national quantitative dietary intake and market data, rather than on expert opinion, pilot tested, and continuously updated for each country, given the rapid transition of dietary patterns globally. Nevertheless, as was evident from Hanley-Cook et al. (2024), a large share of error from brief instruments is attributable to respondent biases [12]. Hence, future studies might also explore whether the magnitude of respondent biases from MDD-W questionnaire are systematic. These findings might allow an informed judgement of whether MDD-W estimates across countries are equally inaccurate, and therefore comparable for global monitoring.

To increase the precision of MDD-W estimates from the GWP, future research should further assess whether MDD-W is also a valid indicator for micronutrient adequacy among males aged 15–49 years across contexts [16, 46]. This might allow for the entire GWP sample of ~ 1,000 adults to be exploited for global monitoring and enable valid comparisons of MDD-W prevalence across sexes. Moreover, given the limited data collected from females aged 15–49 years in certain GWP rounds (e.g., 182 for Switzerland 2023), reporting a moving average of MDD-W from multiple GWP rounds might be more appropriate, as is done for Sustainable Development Goal indicator 2.1.2: Prevalence of moderate and severe food insecurity in the population, based on the Food Insecurity Experience Scale. In any case, national MDD-W prevalence estimates from a single 24-hour recall, which fail to account for day-to-day variability, are likely to be an under- or overestimate of usual MDD-W prevalence depending on whether the population mean dietary diversity is below or above five food groups (i.e., due to extreme values regressing to the mean) [47].

This study has several limitations that should be considered. First, the discordance between sentinel foods on the DHS questionnaire and DQQ is possibly an overestimate. To clarify, when one questionnaire listed a generic food item descriptor such as “milk” and the other listed “curdled milk,” “fermented milk,” and “powdered milk” these were deemed as food items that disagreed, while one might argue that at least one or all three agree. Second, our simulations made the untestable assumption that all respondent biases were carried over from the non-quantitative DQQ to the quantitative 24-HR enumerated in series on the same day, whereas inherent difference in these data collection instrument may influence respondent biases in distinct ways. Third, the two non-inferiority studies were conducted at different time points and in different locations in Ethiopia, so the observed magnitude of errors might not truly be comparable. Lastly, the research team did not have access to the micro-data used in Uyar et al. (2023) [13]. Therefore, simulations comparing sentinel list-based 24-HRs to WFRs could not be run.

In conclusion, defining both the preferred brief instrument and standard data collection platform to be used for a healthy diet indicator might facilitate its uptake in current and forthcoming global monitoring frameworks. In addition, setting international standards for healthy diet metrics would simplify cross-country comparisons, help mitigate biased inferences of change over time within countries, help prevent selective reporting of national statistics, and ultimately improve assessment of global progress on healthy diets. Therefore, for reporting purposes, a hierarchy of nationally-representative country-year data sources was advanced in a recent proposal to include MDD-W as an additional indicator for SDG 2 – starting from quantitative 24-HRs through to MDD-W questionnaires collected through nutrition surveys, DHS, and GWP. Lastly, pilot testing and continuous amendment and improvement of context-specific food lists is crucial to ensure the accuracy and precision of food list-based questionnaires, such as those used to collect the data required for MDD-W.

## Electronic supplementary material

Below is the link to the electronic supplementary material.

Supplementary Material 1

## Data Availability

Nationally representative data and brief data collection instruments supporting the reported results are publicly available on The Demographic and Healthy Surveys Program website (https://dhsprogram.com/, both micro- and meta-data) and Diet Quality Questionnaire Dataverse website (https://dataverse.harvard.edu/dataverse/dqqdata, meta-data only), while meta-data and brief data collection instruments from the non-inferiority studies are publicly available in Hanley-Cook et al. (2020, 2024) [11, 12] and Uyar et al. (2023) [13].

## Abbreviations

24-HR: 24-Hour recall
AIF: Absolute inflation factor
CI: Confidence interval
DHS: Demographic and Health Survey
DQQ: Diet Quality Questionnaire
FAO: Food and Agriculture Organization of the United Nations
HDMI: Healthy Diets Monitoring Initiative
GWP: Gallup World Poll
MDD-W: Minimum Dietary Diversity for Women
pp: Percentage points
RDF: Relative deflation factor
RIF: Relative inflation factor
SD: Standard deviation
WFR: Weighed food record

## Footnotes

1 Personal communication with Dr. Anna Herforth for the Sierra Leone 2021 round. At the time of analysis, only the aggregated statistics (i.e., mean and CI) for the three GWP rounds conducted between 2021 and 2023 were publicly available (*n* = 1,412). Individual-level microdata for each round has since been published on the DQQ Dataverse website (28).

2 At the time of writing, data collection for the 2024–2025 DHS Ethiopia was ongoing. Therefore, the agreement between the DHS and DQQ food lists could not be assessed, as the former is published as part of the final report. Consequently, no speculations as to whether these findings are also relevant to the DHS Ethiopia have been made.
