# Supplementary material for: Minimum Dietary Diversity for Women: precision of national surveys and accuracy of brief data collection instruments
Source: BMC Nutr. 2025 May 27;11:104. doi: 10.1186/s40795-025-01065-7 (PMC12108042; doi:10.1186/s40795-025-01065-7)
Supplement: Supplementary file 1 — Supplementary Material 1 [file 40795_2025_1065_MOESM1_ESM.docx]

|  | **Weighed food record** |
| --- | --- |
|  | **≥15g**  ***n* (%)** |
|  | **All starchy staple foods** |
| List-based recall | 0 |
| Open recall | 0 |
|  | **Beans, peas, and lentils** |
| List-based recall | 9 |
| Open recall | 7 |
|  | **Nuts and seeds** |
| List-based recall | 0 |
| Open recall | 0 |
|  | **Dairy** |
| List-based recall | 0 |
| Open recall | 0 |
|  | **Flesh foods** |
| List-based recall | 1 |
| Open recall | 3 |
|  | **Egg** |
| List-based recall | 1 |
| Open recall | 3 |
|  | **Dark green leafy vegetables** |
| List-based recall | 3 |
| Open recall | 6 |
|  | **Vitamin-A rich fruits and vegetables** |
| List-based recall | 15^1^ |
| Open recall | 16 |
|  | **Other vegetables** |
| List-based recall | 8 |
| Open recall | 10 |
|  | **Other fruits** |
| List-based recall | 6^2^ |
| Open recall | 7 |

**Supplemental table 1. Non-pregnant females underreporting Minimum Dietary Diversity for Women (MDD-W) food groups measured by list-based or open 24-hour recalls, as compared to weighed food record in Ethiopia (*n*=431)**

^1^2× ripe mango, which was not included as an example on the extensive food list. Hence, only 13 observations can be attributed to respondent biases.

^2^1× lemon, which was not included as an example on the extensive food list. Hence, only 13 observations can be attributed to respondent biases.

**Supplemental table 2. Estimated discordance of Minimum Dietary Diversity for Women (MDD-W) prevalence from non-quantitative open and list-based 24-HRs in Ethiopia, when accounting for the proportion of errors attributable to respondent biases to weighed food records**

| **Indicator** | **24-HR % (*n*=969)^1^** | **DQQ % (*n*=969)^1^** | **Percentage point difference between 24-HR and DQQ^1^** | **Open recall bias-adjusted WFR % (*n*=431)^2^** | **Open 24-HR % (*n*=431)^3^** | **Percentage point difference between adjusted WFR and open 24-HR** | **List-based recall bias-adjusted WFR % (*n*=431)^4^** | **List-based 24-HR % (*n*=431)^3^** | **Percentage point difference between adjusted WFR and list-based 24-HR** |
| --- | --- | --- | --- | --- | --- | --- | --- | --- | --- |
| MDD-W | 1.3 | 7.4 | 6.1 | 10.2 (7.7-13.5) | 11.6 (8.9-15.0) | 1.4 (1.2-1.5) | 12.8 (9.9-16.3) | 15.6 (12.4-19.3) | 2.8 (2.5-3.0) |

^1^Point estimates published by Uyar *et al.* (2023) (3). 24-HR, 24-hour recall; NA, not applicable; WFR, weighed food record.

^2^Adjusted by recoding WFR micro-data of individuals that reported consuming a food group during the open 24-HR, when the WFR objectively indicated no food items belonging to the respective food group were consumed, as consumers and individuals that reported not consuming a food group, when the WFR objectively indicated food items belonging to the respective food group were consumed, as non-consumers.

^3^Point estimates published by Hanley-Cook *et al.* (2024) (2), while the 95% confidence intervals were calculated by the authors.

^2^Adjusted by recoding WFR micro-data of individuals that reported consuming a food group during the (extensive) list-based 24-HR, when the WFR objectively indicated no food items belonging to the respective food group were consumed, as consumers and individuals that reported not consuming a food group, when the WFR objectively indicated food items belonging to the respective food group were consumed, as non-consumers.
